# Hydrogen-enabled microstructure and fatigue strength engineering of titanium alloys

**DOI:** 10.1038/srep41444

**Published:** 2017-02-01

**Authors:** James D. Paramore, Zhigang Zak Fang, Matthew Dunstan, Pei Sun, Brady G. Butler

**Affiliations:** 1 Department of Metallurgical Engineering, University of Utah, 135 South 1460 East Room 412, Salt Lake City, Utah 84112, USA; 2 Lightweight and Specialty Metals Branch, United States Army Research Laboratory, 4600 Deer Creek Loop RDRL-WMM-F Aberdeen Proving Ground, Maryland 21005, USA

## Abstract

Traditionally, titanium alloys with satisfactory mechanical properties can only be produced via energy-intensive and costly wrought processes, while titanium alloys produced using low-cost powder metallurgy methods consistently result in inferior mechanical properties, especially low fatigue strength. Herein, we demonstrate a new microstructural engineering approach for producing low-cost titanium alloys with exceptional fatigue strength via the hydrogen sintering and phase transformation (HSPT) process. The high fatigue strength presented in this work is achieved by creating wrought-like microstructures without resorting to wrought processing. This is accomplished by generating an ultrafine-grained as-sintered microstructure through hydrogen-enabled phase transformations, facilitating the subsequent creation of fatigue-resistant microstructures via simple heat treatments. The exceptional strength, ductility, and fatigue performance reported in this paper are a breakthrough in the field of low-cost titanium processing.

The high specific strength and excellent corrosion resistance of titanium alloys have profound implications for sustainability if made economical for widespread commercial utilization. This would not only significantly improve the energy efficiency in applications such as the automotive industry and power generation through lightweighting of high-strength components, but these components would also have significantly increased service life^1^,^2^. To date, the energy-intensive processing routes compulsory for producing high-performance titanium alloys via conventional wrought processing make them unfeasible for most commercial applications, outside of aerospace and biomedicine^2^. Therefore, there is a strong impetus for reducing the embodied energy of titanium components without sacrificing the properties achieved through traditional processes.

Powder metallurgy (PM) has long been sought as means to reduce the embodied energy of titanium components, owing to its near-net-shape (NNS) capabilities. However, PM titanium has traditionally been faced with a trade-off between poor properties or energy-intensive processing^3^. The sintering of blended elemental/master-alloy (BE/MA) powder can produce titanium alloy components at drastically reduced cost. However, the mechanical properties of the BE/MA method are unsatisfactory for demanding applications, due primarily to their poor fatigue strength. By using expensive pre-alloyed (PA) powder and pressure-assisted sintering (e.g. hot isostatic pressing), or by incorporating post-sintering thermomechanical processing (TMP), PM titanium alloys have previously been produced with properties on par with wrought titanium^4^,^5^. However, either route compromises the economic benefit of PM^3^. By using TiH_2_ powder as the feedstock, strides have been made in recent decades towards improving the density and purity of titanium alloys produced by vacuum sintering of relatively inexpensive BE/MA powder^6^,^7^,^8^,^9^,^10^,^11^,^12^,^13^,^14^. However, these alloys still have the coarse lamellar microstructure that is typical of PM Ti-6Al-4V, which is detrimental to mechanical properties, particularly fatigue strength^15^.

## Hydrogen sintering and phase transformation process

The hydrogen sintering and phase transformation (HSPT) process is a new approach in PM processing of titanium alloys^16^,^17^. We have found that utilizing a dynamically controlled hydrogen partial pressure during sintering facilitates the formation of an ultrafine-grained (UFG) microstructure in the as-sintered state via hydrogen-enabled phase transformations. Additionally, we have found that the as-sintered microstructure may be further evolved via simple heat treatments to produce a range of globularized, bi-modal, and lamellar microstructures through non-traditional mechanisms^18^. Conventionally, achieving such microstructures and corresponding mechanical properties in α + β titanium alloys, such as Ti-6Al-4V, requires energy-intensive TMP to produce a driving force for recrystallization^3^. However, the microstructural engineering presented in this paper is achieved without recrystallization during the heat treatments. Fine microstructures, especially globularized/equiaxed and bi-modal microstructures, are known to have excellent fatigue strength in Ti-6Al-4V alloys^19^.

HSPT has been intentionally developed to use only low-energy and commercially-vetted PM processes, such as cold compaction and pressureless sintering. In addition to providing a novel means for producing high-performance titanium alloys, the knowledge gained from this research could provide a baseline to develop processes for similar hydride-forming metal systems, such as magnesium and zirconium alloys. Furthermore, these findings could have significant implications in powder-based additive manufacturing, for which titanium is of particular interest^20^.

The feedstock for HSPT is BE/MA powder containing hydrogenated titanium (TiH_x_), commercially pure titanium (CP-Ti), and/or alloying elements in the form of elemental powder or master alloy. In this study, TiH_2_ powder was milled to a < 37 μm (−400 mesh) particle size via traditional ball milling and mechanically blended with alloying elements. For convenience, the data presented in this paper were generated from cylindrical powder compacts produced via cold isostatic pressing (CIP). However, we have previously shown that uniaxial die pressing is equally effective at producing quality parts via HSPT^21^.

**Figure 1:**
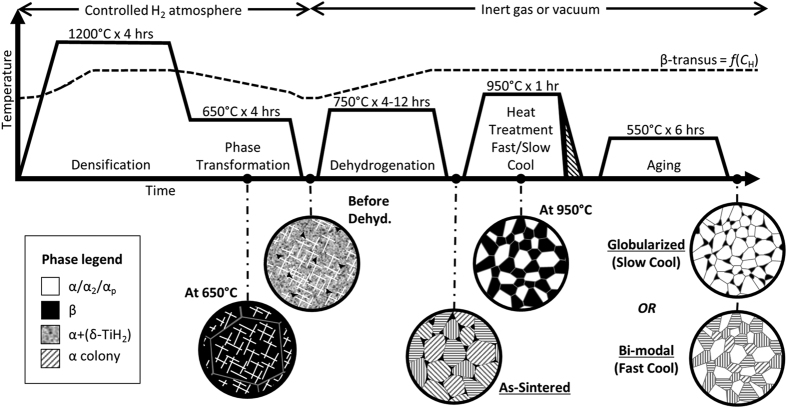
Schematic of the thermal profile, microstructural evolution, and the β-transus (as a function of hydrogen content) during HSPT and subsequent heat treatments to produce Ti-6Al-4V with bi-modal and globularized microstructures. Densification is achieved during β-phase sintering step, activated by the presence of hydrogen. Homogeneous precipitation of low-temperature phases, enabled by the presence of hydrogen, forms an ultrafine-grained (UFG) acicular microstructure of α + α_2_ + β + (δ-TiH_2_) during phase transformation. This is then transformed during dehydrogenation into an UFG structure of α + β. During heat treatment, β grains grow to accommodate the increased equilibrium phase fraction and the UFG α colonies coalesce to form globularized α_p_, which is driven by grain boundary energy. If the alloy is cooled slowly, the β grains recede to the low temperature phase fraction, leaving a fully globularized microstructure. If the material is cooled quickly, the β grains transform into either lamellar or acicular α colonies or martensite, depending on cooling rate, leaving a bi-modal microstructure.

After compaction, the samples were sintered via the HSPT process. Figure 1 is a schematic representation of the thermal profiles used and the microstructural evolution during each step. The hydrogen-free β-transus of Ti-6Al-4V is 995 °C^22^. However, hydrogen is a strong β stabilizer. Therefore, the β-transus varies during the thermal cycles as a function of hydrogen concentration (*C*_H_), as represented by the dashed line in Fig. 1. After sintering, heat treatments were conducted on the HSPT samples according to standard heat treating procedures for wrought-processed Ti-6Al-4V alloys^22^. However, we show that, in contrast with traditional processing, the globularized and bi-modal microstructures were formed through unique mechanisms without TMP or recrystallization.

To eliminate discrepancies caused by other contributing factors and provide a fair comparison of mechanical properties based on microstructure alone, we prepared Ti-6Al-4V via a conventional vacuum sintering process as well. The vacuum-sintered samples were prepared using identical feedstock, powder preparation, and compaction techniques as those used for the HSPT samples in this study. These samples were sintered using only the first step of the HSPT process (1200 °C for 4 hours). However, sintering was performed under high vacuum (<10^−3^ Pa). We have previously demonstrated that heat treatments are ineffective on the coarse vacuum-sintered microstructure^18^. Therefore, the vacuum-sintered samples in this study were not heat treated.

After sintering and before heat treatment, several samples in this study were processed with gaseous isostatic forging (GIF), also known as pneumatic isostatic forging (PIF). This process was used to close the remaining ~1 vol% porosity in both the HSPT and vacuum-sintered samples that remained after sintering. GIF is a low-energy and NNS-compatible process, which is currently used commercially as a means to heal defects and close porosity in low-cost cast^23^ or PM parts^24^,^25^,^26^,^27^,^28^. The samples were processed via GIF by pre-heating in a conventional furnace to ~850 °C and then loaded into a pressure cell, which was rapidly pressurized to ~200 MPa^29^. We have found that GIF is capable of consistently producing Ti-6Al-4V with > 99.9% theoretical density (4.426 g/cc). Additionally, the moderate temperature and short processing time of GIF does not sacrifice the ability of the HSPT material to form wrought-like microstructures via simple heat treatments^30^.

## Results and Discussion

### Fatigue performance and tensile properties of HSPT Ti-6Al-4V

**Figure 2:**
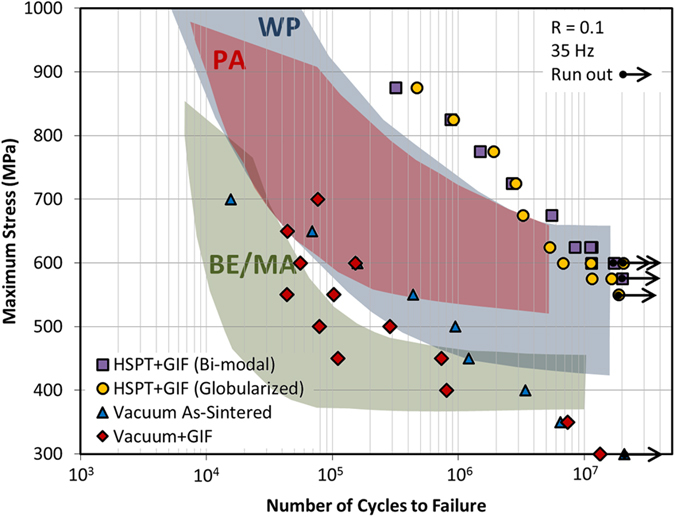
Fatigue performance (S-N) curves for Ti-6Al-4V produced in this study via HSPT and gaseous isostatic forging (GIF) with subsequent heat treatment to produce bi-modal and globularized microstructures, as well as conventional vacuum sintering with and without GIF. Scatter bands are superimposed on the S-N plot for the fatigue performance of Ti-6Al-4V, as reported in the literature^4^,^5^, produced via traditional blended elemental/master-alloy (BE/MA) powder processing, pre-alloyed (PA) powder processing, and wrought processing (WP). As shown, the HSPT process produces fatigue performance far beyond what is typically achievable via BE/MA processing and is competitive with high-performance wrought Ti-6Al-4V. This is a breakthrough in the field, as this is first process to achieve fatigue performance at this level without resorting to costly feedstock materials or processing methods.

**Figure 3:**
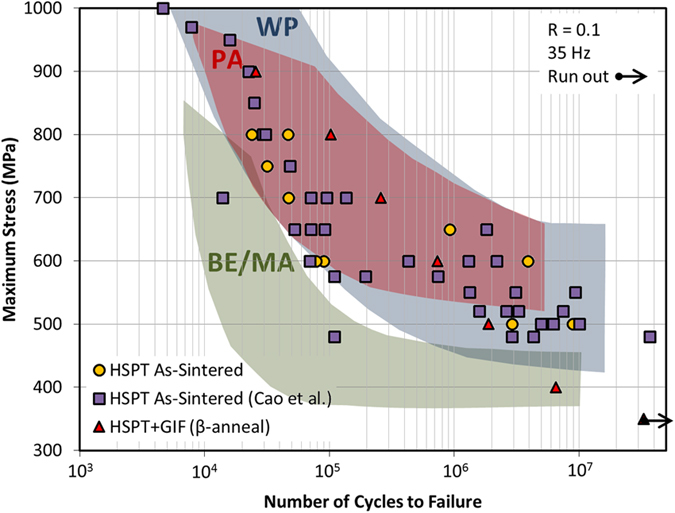
Fatigue performance (S-N) curves for HSPT Ti-6Al-4V in the as-sintered state (produced in this study and as reported by Cao *et al*.^32^) and HSPT Ti-6Al-4V produced in this study after gaseous isostatic forging (GIF) and subsequent heat treatment to produce a β-annealed (coarse lamellar) microstructure. Scatter bands are superimposed on the S-N plot for the fatigue performance for Ti-6Al-4V, as reported in the literature^4^,^5^, produced via traditional blended elemental/master-alloy (BE/MA) powder metallurgy, pre-alloyed (PA) powder metallurgy, and wrought processing (WP).

The most promising results produced in this study are the fatigue performance data for HSPT Ti-6Al-4V. Figure 2 shows the fatigue performance (S-N plots) of Ti-6Al-4V produced in this study via HSPT with GIF and heat treatment, as well as vacuum sintering with and without GIF. As per the ASTM E466 standard^31^, fatigue testing was done using cyclic axial loading with constant amplitude on smooth bar (polished) specimens. For reference, scatter bands have been superimposed on Fig. 2 showing the literature-reported fatigue performance of Ti-6Al-4V produced via BE/MA and PA powder metallurgy, as well as wrought-processing (WP)^4^,^5^. As shown, HSPT is able to produce Ti-6Al-4V with fatigue properties that far exceed what is typical for BE/MA Ti-6Al-4V without incorporating costly post-sintering processing to improve fatigue performance. Additionally, the HSPT fatigue strengths are competitive with the upper end of wrought processing. The bi-modal samples exhibited the highest 10^7^ cycle fatigue strength (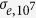 ≈ 600 MPa), followed by the globularized microstructure (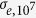 ≈ 575 MPa). The as-sintered HSPT microstructure without GIF or heat treatment has been previously reported to have a 10^7^ cycle fatigue strength of 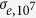
≈500 MPa^32^. S-N plots for as-sintered HSPT Ti-6Al-4V as previously reported and produced in this study are provided in Fig. 3. Additionally, S-N plots for HSPT Ti-6Al-4V that has been subsequently β-annealed to produce a coarse lamellar structure is also shown in Fig. 3.

As expected, vacuum sintering produced fatigue performance that fell largely within the scatter bands of traditional BE/MA Ti-6Al-4V. It has long been known that fatigue cracks can readily propagate across lamellar α colonies^15^. Therefore, the coarse size of the α colonies in the vacuum-sintered microstructure dominated the high cycle fatigue performance of these samples. As such, closing the ~1 vol% porosity via GIF had little effect on the fatigue performance of the vacuum-sintered samples. Therefore, the vacuum-sintered samples exhibited the same relatively poor fatigue performance in both the as-sintered and as-GIF’d conditions (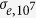 ≈ 300 MPa).

**Figure 4:**
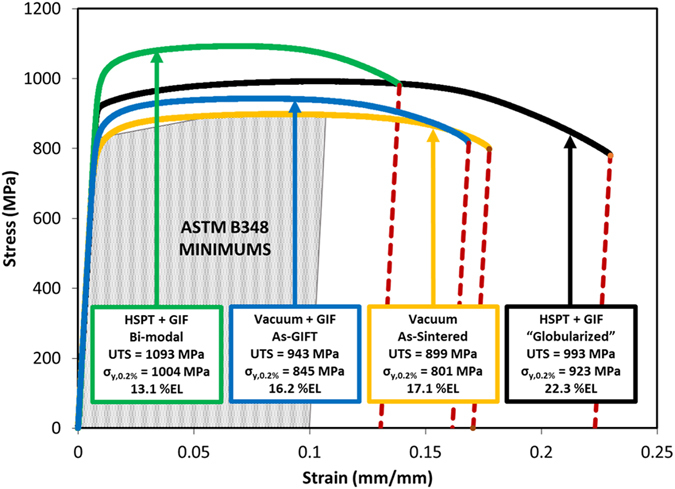
Tensile stress-strain curves for Ti-6Al-4V produced via HSPT and gaseous isostatic forging (GIF) with subsequent heat treatment to produce bi-modal and globularized microstructures, as well as conventional vacuum sintering with and without GIF. The shaded area of the stress-strain plot indicates the minimum strength and ductility for wrought Ti-6Al-4V by the ASTM B348 standard^43^.

Representative engineering stress-strain curves of Ti-6Al-4V produced by HSPT and vacuum sintering are shown in Fig. 4. Additionally, the average mechanical properties for all conditions discussed in this paper are given in Table 1. For reference, the ASTM B348 standard for wrought Ti-6Al-4V is given, which is also represented by the shaded area in Fig. 4. As shown, the HSPT process is capable of producing a range of both strength and ductility well beyond the minimums set forth by the ASTM standard. Therefore, the mechanical properties produced via this process may be tuned for application-specific requirements. Furthermore, these values are very competitive with those reported in the literature for state-of-the-art wrought Ti-6Al-4V^19^. The bi-modal microstructures exhibited the greatest tensile strength, approximately 1100 MPa, while the globularized samples had the greatest ductility, exceeding 21%EL and 45%RA. While the coarse lamellar microstructure produced by vacuum sintering exhibited good ductility, the strength was significantly lower than that produced by HSPT. As shown, the 0.2% offset yield strength produced by vacuum sintering was just above the ASTM standard in the as-GIF’d condition, and fell below the ASTM standard in the as-sintered condition.

The effect of microstructure on fatigue performance is further elucidated when comparing the ultimate tensile strength to the fatigue strength of these materials. As shown in Table 1, the fatigue strength of the HSPT bi-modal and globularized microstructures was approximately 54 and 57% of the ultimate tensile strength, respectively. For comparison, the coarse-grained vacuum-sintered microstructures had fatigue strengths of approximately 33 and 32% of the ultimate tensile strength in the as-sintered and as-GIF’d conditions, respectively. Therefore, even when normalized for strength, the HSPT-produced structures have significantly improved fatigue resistance.

### Microstructural evolution during sintering and dehydrogenation

The exceptional mechanical properties that are available via the HSPT process result from the use of hydrogen during the sintering process. The presence of dissolved hydrogen during sintering is known to significantly improve densification during sintering of titanium alloys by increasing self-diffusion kinetics of β-Ti^7^. Therefore, densification to over 99% theoretical density (%TD) is achieved during the super-transus densification step, during which the material is entirely β-T(H) (β phase with dissolved hydrogen). In order to clearly understand the phase transformations during HSPT, in situ synchrotron X-ray diffraction (XRD) was performed using a custom-designed sapphire tube furnace installed in the synchrotron beamline 11-ID-C at the Advanced Photon Source, Argonne National Laboratory. We have previously published detailed descriptions of the phase transformations during the sintering process as well as an improved (Ti-6Al-4V)-H pseudo-binary phase diagram^33^,^34^.

After densification, the material is cooled to a sub-transus temperature and dwelled during the phase transformation step (Fig. 1). During this step and subsequent cooling, the presence of β-stabilizing hydrogen (~1.2 wt%) causes the (Ti-6Al-4V)-H alloy to act as a metastable β alloy^34^. This allows the microstructure to be refined via phase transformations not possible in hydrogen-free Ti-6Al-4V, which is otherwise an α + β alloy. In particular, these phase transformations are homogenous precipitation of the α_2_-Ti_3_Al (ordered HCP) and α-Ti (HCP) phases, which results from a spinodal decomposition of the β phase during the dwell. This is followed by further decomposition of the β phase to α and δ-TiH_2_ via a eutectoid reaction upon cooling to room temperature^34^.

**Figure 5:**
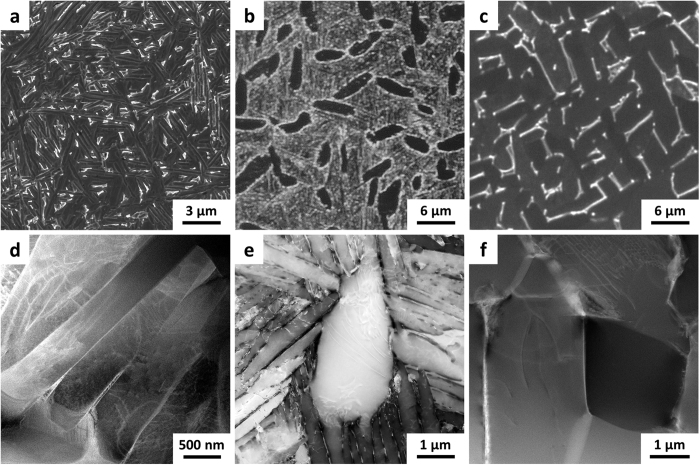
Secondary electron scanning electron micrographs (SE/SEM) and high angle annular dark-field scanning transmission electron micrographs (STEM) of Ti-6Al-4V produced via the HSPT process. (**a**) SE/SEM of HSPT as-sintered ultrafine-grained (UFG) microstructure, (**b**) SE/SEM of HSPT as-aged bi-modal microstructure, (**c**) SE/SEM of HSPT as-aged globularized microstructure, (**d**) STEM of UFG α colony in HSPT as-sintered microstructure, (**e**) STEM of HSPT as-aged bi-modal microstructure, and (**f**) STEM of HSPT as-aged globularized microstructure.

During dehydrogenation, the hydrogen-stabilized phases, β-Ti and δ-TiH_2_, transform into α-Ti with retained β (Fig. 1). In Ti-6Al-4V, the presence of ordered α_2_-Ti_3_Al results from saturation of α phase with aluminum^35^. Therefore, the increased α phase fraction resulting from hydrogen removal decreases the average aluminum concentration of this phase, causing the ordered α_2_ phase to transform into α. The resulting as-sintered condition has an UFG α + β microstructure, as shown in Fig. 5-a and d. The α grains (dark contrast in SE/SEM) are present as UFG colonies measuring several microns across. These colonies consist of α grains with lengths equal to the colony width and individual laths of sub-micron dimensions. The β phase (light contrast in SE/SEM) is discontinuously distributed as ultrafine grains, located at the triple points of the α colonies (seen as white spots in Fig. 5-a).

### Microstructural evolution during heat treatment and ageing

**Figure 6:**
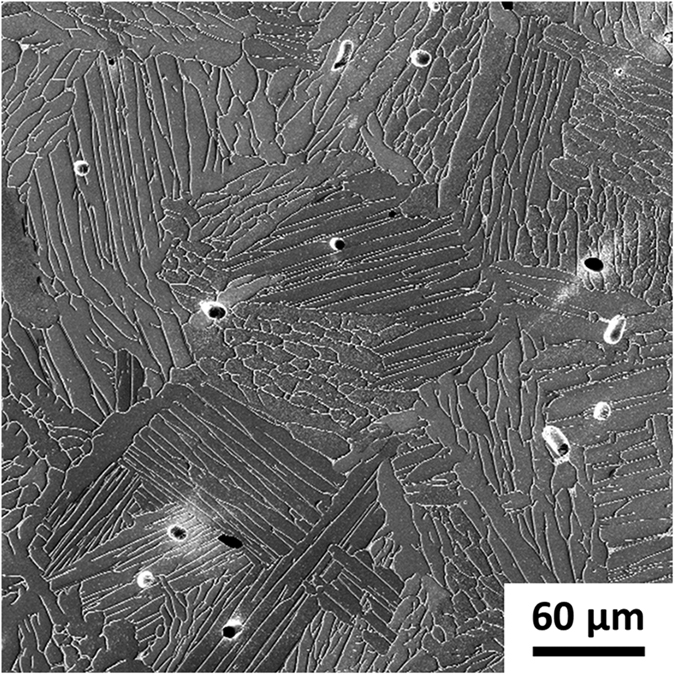
Secondary electron (SE) scanning electron micrograph (SEM) of vacuum-sintered Ti-6Al-4 V. A coarse lamellar microstructure is typical of what is produced by vacuum sintering, which is detrimental to fatigue performance if not refined via thermomechanical processing (TMP).

We present the results from two different heat treatments of the as-sintered HSPT material: sub-transus heat treatment (950 °C for 1 hour) with water quenching to form a bi-modal microstructure (Fig. 5b and e), and sub-transus heat treatment (950 °C for 1 hour) with furnace cooling (~10 °C/min) to form a globularized microstructure (Fig. 5c and f). Super-transus heat treatment may also be performed to form a β-annealed (fully lamellar) microstructure. However, during a super-transus heat treatment, the microstructure is entirely β phase, which forms relatively coarse lamellar α colonies upon cooling similar to that produced via traditional vacuum sintering (Fig. 6). Therefore, only the sub-transus heat treatments with furnace cooling (slow cool) and water quenching (fast cool) are represented in Fig. 1; the shaded area on the thermal profile represents the variable cooling rate.

The key innovation of HSPT is the formation of desirable fatigue-resistant microstructures without requiring deformation and recrystallization (i.e. TMP). We propose two main mechanisms for the novel microstructural evolution of HSPT Ti-6Al-4V during sub-transus heat treatments. First, as the material is heated to 950 °C, the large degree of grain boundary energy drives coalescence of the UFG α colonies into single globularized primary α (α_p_) grains. Second, the size, distribution, and morphology of the β phase in the as-sintered condition enables the formation of equiaxed β grains several microns in diameter and evenly distributed throughout the material. Therefore, we propose that at the heat treatment temperature the microstructure consists of alternating globularized α_p_ and β grains, with roughly the same size and shape (Fig. 1: At 950 °C). According to the (Ti-6Al)-V pseudo-binary phase diagram^36^, the equilibrium phase fractions at this temperature are ~60 vol% β and ~40 vol% α.

**Figure 7:**
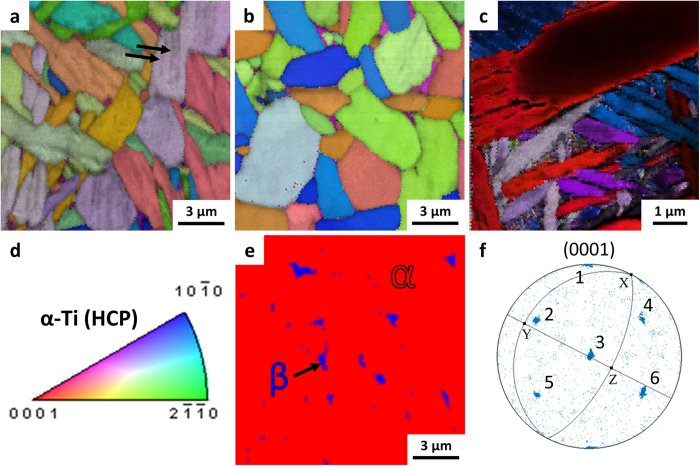
Electron backscatter diffraction (EBSD) and transmission Kikuchi diffraction (TKD) data of Ti-6Al-4V produced via the HSPT process. (**a**) EBSD of as-sintered ultrafine-grained (UFG) microstructure, (**b**) EBSD of globularized microstructure produced via sub-transus heat treatment with furnace cooling and ageing, (**c**) TKD of bi-modal microstructure produced via sub-transus heat treatment with water quenching and ageing, (**d**) color legend for grain orientation of α phase in (**a–c**), (**e**) color-coded phase map showing distribution of β phase located at the triple points of the α_p_ grains in the fully globularized microstructure, and (**f**) pole figure of bi-modal microstructure rotated to show all variants of the (110)_β_||(0001)_α_ Burger’s relationship with the sample axes of image (**c**) overlaid on the stereographic projection. Images (**a–c**) are inverse pole figure (IPF) Euler maps with overlaid image quality (IQ) maps to highlight low-angle grain boundaries. It should be noted that the striations (marked by arrows) in (**a**) indicate that the apparent grains in the IPF map are actually colonies of parallel α grains, which coalesce during heat treatment and are, therefore, not visible within the α_p_ grains in image (**b**) or (**c**).

Evidence for these mechanisms are visible in electron backscatter diffraction (EBSD) and transmission Kikuchi diffraction (TKD) micrographs. Figure 7-a through c are composite micrographs produced by EBSD (a and b) or TKD (c) consisting of an inverse pole figure (IPF) Euler map with a superimposed image quality (IQ) map. The IPF map shows the relative orientation of the grains as a color gradient, following the legend given in Fig. 7d. An IQ map is useful for highlighting low-angle grain boundaries, which produce overlapping diffraction patterns and show up as darker contrast. In the as-sintered condition (Fig. 7-a), the IQ map clearly shows striations within each apparent grain (marked with arrows), which are absent in the heat-treated conditions. By comparing Fig. 7a to b and c, it is clear that the as-sintered microstructure contains colonies of UFG α lamellae that coalesce to form globularized α_p_ grains during sub-transus heat treatment. That is, the α_p_ grains in the heat treated microstructures have similar size and shape to the UFG colonies in the as-sintered microstructure. Additionally, the morphology and distribution of the β grains in the globularized microstructure are shown in Fig. 7e, which clearly shows that the β grains (shown in blue) are very fine, discontinuous, and largely confined to the triple points of the α_p_ grains.

Whether a globularized or bi-modal microstructure is formed during a sub-transus heat treatment is determined by the cooling rate^37^. If the material is cooled very slowly, a greater degree of phase transformation occurs at higher temperatures, which minimizes nucleation of new α grains and allows for faster diffusion during the transformation. Therefore, when Ti-6Al-4V is cooled from the heat treatment temperature at a sufficiently slow rate, the β grain boundaries will effectively recede and leave behind α grains with a very low aspect ratio, resulting in a fully globularized microstructure. Conversely, if the material is cooled rapidly, a greater degree of undercooling will exist during the phase transformation, which produces more nucleation of new α grains. The new α grains form with a (110)_β_||(0001)_α_ Burgers relationship with the parent β grains^15^,^37^,^38^. This causes the new α grains to form colonies of parallel grains, resulting in a bi-modal microstructure of globularized α_p_ grains and colonies of lamellar secondary α grains. All 12 variants (6 variants and the corresponding negatives) of this Burger’s relationship are visible in the pole figure shown in Fig. 7f. If the material is cooled fast enough to arrest diffusion (>1000 °C/min for Ti-6Al-4V)^38^ and with sufficient undercooling, the β grains will transform into martensite via a diffusionless transformation.

**Figure 8:**
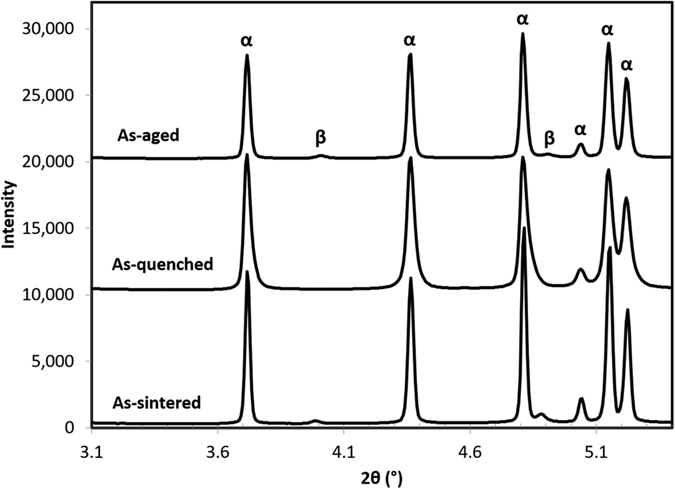
Synchrotron X-ray diffraction patterns of the as-sintered, as-quenched, and as-aged condition in the process of forming Ti-6Al-4V with a bi-modal microstructure. The disappearance of the β peaks in the as-quenched condition and reappearance in the as-aged condition is clear evidence of diffusionless transformation of the β grains into martensite during the water quenching process and subsequent transformation of martensite into α colonies during ageing.

In order to gain a clear understanding of the microstructural evolution resulting from the cooling rate, synchrotron X-ray diffraction (XRD) was performed on HSPT Ti-6Al-4V at different points throughout the heat treatment and ageing processes. These experiments were conducted using the same experimental setup used to study the phase transformations during sintering^33^. Figure 8 shows the synchrotron XRD spectra of Ti-6Al-4V produced via HSPT and heat treated at 950 °C with a subsequent water quench in the as-sintered, as-quenched, and as-aged conditions. As shown in the spectra, there are no β peaks present in the as-quenched condition, though they are visible in both the prior and subsequent conditions. Because vanadium is a strong β stabilizer, diffusional transformations of β to low-temperature phases consistently result in residual β in Ti-6Al-4V. Therefore, the absence of β peaks indicates that β transformed via a diffusionless reaction during quenching to form metastable martensite. Subsequently, the ageing process is sufficient to transform the martensite into α + β colonies, explaining the reappearance of the β peaks after ageing. However, the relatively low ageing temperature (550 °C) results in these grains maintaining an acicular morphology. Therefore, this heat treatment produces a bi-modal structure of globularized α_p_ grains and ultrafine acicular α colonies with retained β (Fig. 5b and e, and Fig. 7c).

The GIF process used in this study served purely as a means to close the residual porosity that remained after sintering, which could obscure the effect of microstructure on mechanical properties. SEM and EBSD evaluation of samples directly after GIF revealed a microstructure consisting of globularized α grains with ultrafine β grains at the triple points, similar to the globularized microstructure of only α and β grains shown in Figs 5c and 7b. Therefore, the elevated temperatures required for the GIF process apparently cause coalescence of the ultrafine α colonies that result from the HSPT sintering process. The GIF applies fully hydrostatic compressive stress well below 1 GPa. Therefore, this process should provide minimal driving force for pressure-induced phase transformations, which are unlikely under this pressure and loading condition^39^,^40^. Furthermore, the fact that GIF was done before heat treatment and that the GIF temperature was ~100 °C lower than the heat treatment temperature, we believe that any temperature or pressure-induced transformations resulting from the GIF process should have been effectively negated by the subsequent heat treatments.

### Crack initiation and propagation during fatigue testing

**Figure 9:**
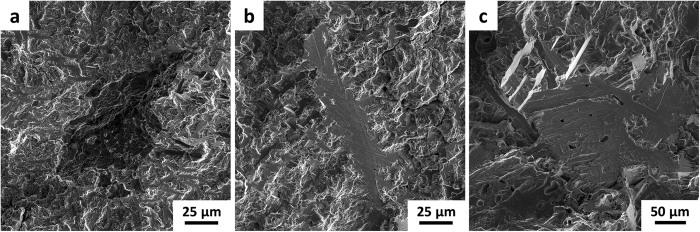
Secondary electron scanning electron micrographs (SE/SEM) of Ti-6Al-4V high cycle fatigue fracture surfaces. (**a**) HSPT as-aged bi-modal microstructure that failed at 11.5 million cycles at 625 MPa, (**b**) HSPT as-aged globularized microstructure that failed at 16.4 million cycles at 575 MPa, and (**c**) vacuum-sintered coarse lamellar microstructure that failed at 3.4 million cycles at 400 MPa. It should be noted that the micron bar in (**c**) has a value approximately twice that of the other two.

Figure 9 shows representative initiation sites on the fracture surfaces of high cycle fatigue samples produced both by HSPT with GIF and heat treatment as well as vacuum sintering. The fractographs of vacuum-sintered Ti-6Al-4V produced in this study were extremely similar for both as-sintered and as-GIF’d material. Therefore, the vacuum-sintered fractograph shown in Fig. 9c is representative of both conditions. As shown, the vacuum-sintered samples consistently have large faceted areas at the initiation site often measuring over 100 μm across, which are similar in width to an entire α colony in the microstructure. This observation is consistent with the literature, in which it has long been reported that fatigue cracks can propagate transgranularly across an entire α colony with little resistance^41^. In contrast, the initiation sites in the HSPT bi-modal (Fig. 9a) and globularized microstructures (Fig. 9b) are significantly different. The faceted regions in these microstructures are similar in dimension with the α_p_ grains or ultrafine α colony widths in either microstructure. Therefore, the refined microstructure produced by the HSPT process significantly reduces the length of relatively unimpeded fatigue crack propagation, resulting in the significantly improved fatigue strength.

It should be noted that an elongated faceted region shown in Fig. 9b is commonly observed at the initiation sites of heat-treated HSPT fatigue samples. It is believed that these faceted regions are the result of relatively large grain boundary α (α_GB_) grains, which have also been observed on polished specimens at low magnification along the prior β grain boundaries. It is believed that the α_GB_ grains form during the dehydrogenation step via heterogeneous nucleation of α grains along the boundaries of the β grains that were present during the sintering step. These α_GB_ grains form due to the changing α/β phase fractions as hydrogen is removed. While the α_GB_ grains apparently served as fatigue crack initiators in several bi-modal and globularized samples, the obvious α_GB_ facets only exist at the initiation site. Therefore, the overall effect on fatigue performance by the α_GB_ grains is not currently known. We are currently working to effectively minimize the occurrence and size of the α_GB_ grains by modifying the dehydrogenation profile, which may further improve the mechanical properties. However, that work is still in progress and beyond the scope of this paper.

## Concluding remarks

In this paper, we have demonstrated a fundamentally new approach to producing Ti-6Al-4V with state-of-the-art properties via low-energy and low-cost processing routes. HSPT utilizes hydrogen-enabled phase transformations in a novel sintering process, which facilitate unique mechanisms for microstructural evolution. Therefore, HSPT is capable of producing NNS Ti-6Al-4V components with wrought-like microstructures and tunable mechanical properties via simple heat treatments that do not require energy-intensive TMP. In particular, we have demonstrated the ability of this process to produce fatigue performance that is on par with state-of-art wrought processing using only low-energy processes.

## Methods

### Powder preparation

Commercially pure (CP) TiH_2_ powder with a 250-841 μm (−20 + 60 mesh) particle size range and 60Al/40 V (60 wt% Al, 40 wt% V) master alloy with a < 44 μm (−325 mesh) particle size was received from Reading Alloys (AMETEK). The as-received TiH_2_ was ball milled in a 304 stainless steel milling jar (97.2 mm ID × 142.9 mm L). The milling jar was loaded with 250 g of TiH_2_ and 2.5 kg of 6.35 mm diameter 440 C stainless steel balls. Milling was performed on a rolling mill for 30 minutes at 64.7 RPM (47% critical speed). After milling, the powder and balls were sieved for 1 hour using 149 μm (100 mesh) and 37 μm (400 mesh) sieves on a dual-drive vibratory sieve shaker with an ultrasonic agitator (HK Technologies HK-8 with Ultrasonic SLPT Deblinding). The < 37 μm (−400 mesh) sieve cut was collected for sample preparation. The < 37 μm TiH_2_ powder was then blended with as-received 60Al/40 V master alloy powder and mixed for 1 hour in a 1 L polyethylene bottle using a tumbling shaker-mixer (Glenn Mills T2C).

### Compaction

To prepare each fatigue/tensile sample blank, 60 g of the blended powder mixture was loaded into latex isostatic pressing bags (Trexler Rubber, 19.05 mm ID × 114.3 mm L). The pressing bags were then evacuated to remove excess air and loaded into a cold isostatic press (CIP, American Isostatic Presses CP360). The samples were then isostatically pressed at 350 MPa for 7 minutes to produce green parts with a cylindrical shape.

### Sintering

Sintering was conducted using a custom-built tube furnace capable of high vacuum ( < 10^−3^ Pa) as well as mixtures of flowing ultrahigh purity (UHP) Ar and H_2_ gases. The vacuum system consisted of a rotary vane roughing/backing pump and an oil diffusion pump (Edwards Diffstak 100/300 M). The HSPT sintering process was performed using the thermal profile shown in Fig. 1. A 50.7 kPa H_2_ partial pressure was used during the sintering and phase transformation step of the HSPT process. The H_2_ partial pressure was produced by flowing 1 L/min of H_2_ and 1 L/min of Ar at atmospheric pressure using electronic mass flow controllers (Aalborg GFC). The dehydrogenation step was conducted under high vacuum. Vacuum sintering was performed using only the first step of the HSPT process (1200 °C for 4 hours) under high vacuum. Sintering and dehydrogenation were conducted in batches of 10 samples.

### GIF process

For the GIF process, the samples are first preheated in a conventional furnace to ~850 °C. To achieve the rapid pressurization of the pressure cell, the pressure cell is connected to a gas reservoir by a valve. The gas reservoir is pressurized to ~415 MPa with argon using a gas booster pump. The preheated samples are loaded into the pressure cell, which is then sealed. Opening the valve causes the pressure cell to be rapidly pressurized to ~200 MPa in several seconds, which is well above the flow stress of the material at this temperature. This creates an “air hammer” that isostatically closes the residual porosity in the samples.

### Heat treatment and ageing

Heat treatment and ageing were conducted in the same furnace used for sintering. Heat treatments with furnace cooling were conducted under high vacuum. Heat treatments with water quenching were conducted under flowing Ar to allow for rapid opening of the furnace. During quenching, samples were unloaded from the furnace and quickly ( < 3 s) submersed in room temperature water. All ageing experiments were conducted under high vacuum. Samples were heat treated and aged in batches of 10 samples.

### Fatigue/tensile bar preparation

Each tensile/fatigue blank was first electric-discharge machined (EDM) to produce a right cylindrical geometry. The samples were then machined on a computer numerical controlled (CNC) lathe to the necessary dimensions. As per the ASTM E8^42^ standard, the tensile bars had a 6.35 mm (0.25”) diameter by 25.4 mm (1”) long gauge and 3/8”-15 threaded grips. As per the ASTM E466^31^ standard, the fatigue bars had a continuous radius gauge with a 6.35 mm (0.25”) minimum diameter and 50.8 mm (2”) radius of curvature, and 12.7 mm (0.5”) diameter smooth grips. For reference, technical drawings of the sample dimensions used in this study have been supplied in the supplementary information accompanying this paper. After machining, the fatigue bars were polished to a mirror finish (smooth bar) on a polishing wheel with buffing compound.

### Fatigue testing

Fatigue tests were performed using an MTS Landmark Servohydraulic Test System capable of up to 120 kN of force and 60 Hz cyclic loading. Control and data acquisition were accomplished using MTS TestSuite software. Fatigue bar grips were unthreaded and loaded directly into hydraulic wedges on the test frame with serrated V-shaped grips. Force amplitude control was used to apply cyclic axial loading at 35 Hz with a 0.1 stress ratio (R = σ_min_/σ_max_) to encourage crack closure between cycles.

### Tensile testing

Tensile tests were performed using the same test frame and software used for fatigue tests. The tensile bars were threaded onto high strength steel rods mounted in the wedge grips of the MTS. Tensile tests were performed at a strain rate of 3 × 10^−4^ s^−1^, as defined by the ASTM standard for testing titanium alloys^43^. Strain was measured using an MTS 25.4 mm (1”) contact extensometer mounted to the gauge.

### Metallographic preparation (SEM/EBSD)

Each sample that was chosen for SEM/EBSD analysis was first sectioned using a precision sectioning saw (Allied High Tech TechCut 5). Care was taken to ensure than an interior surface of each sample was exposed by the sectioning process. This was done by sectioning a cylindrical sample at a length from the sample end that is equal to or greater than the radius of the sample. Sectioning was performed with a 203 mm resin-bonded SiC blade at 3,000 rpm and a cut rate of 5 mm/min. After sectioning, each specimen was mounted in a conductive graphite-impregnated mounting resin using a hot press (Allied High Tech TechPress 2). The samples were then ground and polished using an automatic polisher (Allied High Tech MetPrep 3 with AD-5 automatic fluid dispenser). The samples were first ground with ANSI 180 grit SiC paper until flat, followed by 320 grit SiC paper to remove the coarse scratches. After grinding, the samples were polished using 9 μm followed by 3 μm diamond suspension in oil. The samples were then polished for 5 minutes using an attack polish consisting of 0.04 μm colloidal silica mixed with 30 vol% of H_2_O_2_ (30%). A small area of each polished sample was etched to reveal microstructural details. The etchant used was Kroll’s solution, prepared from concentrated hydrofluoric acid, concentrated nitric acid, and water (2 vol% HF: 3 vol% HNO_3_: 95 vol% H_2_O).

### Metallographic preparation (STEM/TKD)

To prepare samples suitably thin for STEM/TKD, the samples were first sectioned into discs approximately 50.8 mm in diameter and 250 μm thick. Several small discs were then taken from each section using a 3 mm hole punch. After punching, the 3 mm discs were pre-thinned equally on each side using a thickness-controlled specimen grinder (Fischione Model 160) to approximately 100 μm. This was accomplished on a polishing wheel using ANSI 600 grit SiC paper to remove the damage layer caused by sectioning, followed by 800 grit and then 1200 grit SiC paper to produce a polished surface. Final thinning was performed using an automatic twin-jet electropolisher (Fischione Model 120 Power Controller and Model 220 Low-Temp Container). The electropolisher is equipped with an optical sensor, which will automatically stop polishing when perforation of the sample is detected as light penetrating the thinned section. The electrolyte used for electropolishing was prepared from concentrated perchloric acid (60%), 2-butoxyethanol, and methanol (6 vol% HClO_4_: 34 vol% BuOC_2_H_4_OH: 60 vol% MeOH). Electropolishing was conducted at −30 °C with a high electrolyte flow rate and a 35 V polishing potential, which corresponded to an approximately 10 mA polishing current.

### Microscopy

SEM micrographs were acquired with an FEI NovaNano using a through the lens detector (TLD) at 2.0 kV and a 5 mm working distance (WD). EBSD and TKD data were acquired with an EDAX Hikari EBSD camera and EDAX TEAM EBSD Analysis System software. EBSD was acquired at 20 kV and an 8 mm WD with the sample tilted to 70°. TKD was acquired at 30 kV and a 2 mm WD with the sample tilted to −20° on a custom stage. STEM micrographs were acquired with a JEOL 2100 F TEM using a high angle annular dark-field detector (HAADF) in STEM mode at 200 kV and a probe diameter of 0.2 nm.

### Synchrotron XRD

Synchrotron XRD experiments were performed on the 11-ID-C beam-line at the Advanced Photon Source, Argonne National Laboratory, with high-energy (115 keV, wavelength = 0.1080 Å) X-ray illumination. The energy of the synchrotron X-ray source at 11-ID-C is sufficiently high to penetrate through the samples. XRD patterns were collected using a 2-D detector as a series of rings. The 2-D diffraction patterns were converted to XRD spectra using Fit2D software.

## Additional Information

**How to cite this article**: Paramore, J. D. *et al*. Hydrogen-enabled microstructure and fatigue strength engineering of titanium alloys. *Sci. Rep.* **7**, 41444; doi: 10.1038/srep41444 (2017).

**Publisher's note:** Springer Nature remains neutral with regard to jurisdictional claims in published maps and institutional affiliations.

## Supplementary Material

Supplementary Information

## Figures and Tables

**Table 1:** Average mechanical properties of Ti-6Al-4V produced in this study via HSPT and traditional vacuum sintering. The ASTM B348 standard for Grade 5 Ti-6Al-4V^43^ is given for reference.

Condition	Microstructure	UTS (MPa)	σ_y,0.2%_ (MPa)	%EL	%RA	E (GPa)	σ_e,10^7^_ (MPa)	σ_e_/UTS (%)
HSPT As-Sintered	UFG Lamellar	1018	963	14.2	30.1	111	500*	49
HSPT + GIF + HT	Bi-modal	1101	1019	13.6	27.3	117	600	54
HSPT + GIF + HT	Globularized	1002	931	21.4	45.0	118	575	57
Vacuum As-Sintered	Coarse Lamellar	901	801	17.9	32.3	114	300	33
Vacuum + GIF	Coarse Lamellar	940	840	16.5	32.1	116	300	32
ASTM B348 (min.)	—	895	828	10.0	25.0	—	—	—

^*^Fatigue strength of as-sintered HSPT Ti-6Al-4V as reported by Cao *et al*.^32^.
