# Supplementary material for: Hydrogen-enabled microstructure and fatigue strength engineering of titanium alloys
Source: Sci Rep. 2017 Feb 1;7:41444. doi: 10.1038/srep41444 (PMC5286406; doi:10.1038/srep41444)
Supplement: Supplementary Information [file srep41444-s1.pdf]

Hydrogen-enabled microstructure and fatigue strength engineering of titanium alloys

James D. Paramore [1,2]*, Zhigang Zak Fang [1]*, Matthew Dunstan [1], Pei Sun [1], and Brady G. Butler [2]

[1] Department of Metallurgical Engineering, University of Utah, Salt Lake City, Utah, USA

[2] Lightweight and Specialty Metals Branch, United States Army Research Laboratory, Aberdeen Proving Ground, Maryland, USA

Supplementary Information

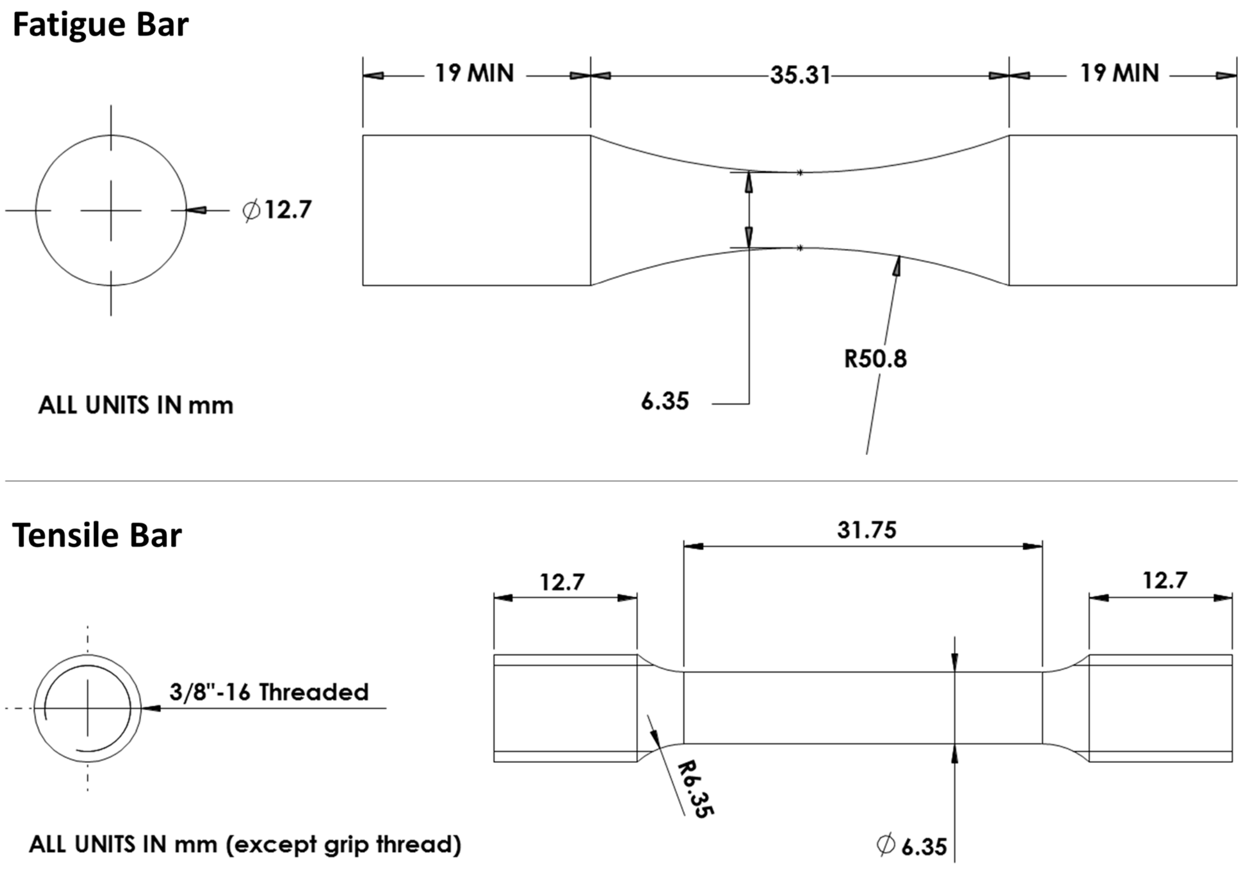

Supplementary Figure 1: Dimensions of fatigue and tensile bars used in this study.
